# Health Self-Management Applications in the Work Environment: The Effects on Employee Autonomy

**DOI:** 10.3389/fdgth.2020.00009

**Published:** 2020-07-17

**Authors:** Anne Bonvanie, Manda Broekhuis, Onne Janssen, Els Maeckelberghe, J. (Hans) C. Wortmann

**Affiliations:** ^1^Department of Operations, Faculty of Economics and Business, University of Groningen, Groningen, Netherlands; ^2^Department of Human Resource Management and Organizational Behaviour, Faculty of Economics and Business, University of Groningen, Groningen, Netherlands; ^3^Institute for Medical Education, University Medical Center Groningen, University of Groningen, Groningen, Netherlands

**Keywords:** health self-management, autonomy, wearables, sensor technology, work place health promotion

## Abstract

Organizations increasingly use Health Self-Management Applications (HSMAs) that provide feedback information on health-related behaviors to their employees so that they can self-regulate a healthy lifestyle. Building upon Self-Determination Theory, this paper empirically investigates the basic assumption of HSMAs that their self-management feature provides employees with autonomy to self-regulate their health-related behavior. The two-phase experimental study contained a 4-weeks HSMA intervention in a healthcare work environment with a feedback factor (performance vs. developmental) and pretest and posttest measurements of participants' perceived autonomy. Following the experiment, interviews were conducted with users to gain an in-depth understanding of the moderating roles of feedback and BMI (a proxy for health) in the effects of HSMA on perceived autonomy. Findings reveal that the use of an HSMA does not significantly increase perceived autonomy, and may even reduce it under certain conditions. Providing additional developmental feedback generated more positive results than performance feedback alone. Employees with higher BMI perceived a greater loss of autonomy than employees with lower BMI. The reason for this is that higher-BMI employees felt external norms and standards for healthy behavior as more salient and experienced more negative emotions when those norms are not met, thereby making them more aware of their limitations in the pursuit of health goals.

## Introduction

To increase overall productivity and decrease workforce costs, organizations are increasingly embracing workplace health promotion programs as a critical strategy for improving employee health and work outcomes (1, 2). These programs tend to focus on individual health factors, such as diet and physical exercise, and represent a broad range of disease prevention, and health promotion methods such as health checks (3), gym subscriptions (1), physical activity [e.g., (4–6)], and vitality training (2). A common denominator in health promotion programs is an increasing reliance on health self-management applications (HSMAs) that provide individual users with key metrics about their bodily functioning and personal health-related behaviors (7, 8). For example, wearable activity trackers are used to inform users about the number of steps they take, the number of stairs they climb, and the intensity levels of their physical activities on a daily basis [e.g., (4)].

A core assumption underlying the use and usefulness of such HSMAs is that their self-management feature provides employees with autonomy and control to self-regulate their health-related behavior. Specifically, derived from Self-Determination Theory (SDT) (9, 10), the notion is that the use of HSMAs promotes a sense of autonomy through which employees become intrinsically and deeply engaged in self-regulating their behavior. Critical elements for behavioral change and health improvements are monitoring, goal setting, and action planning (2, 7, 8, 11). However, although a substantial body of research has shown the potential of HSMAs in promoting employee health (4, 12), no empirical studies have examined and proven the basic assumption that HSMAs increase employees' perceptions of autonomy in the self-regulation of their health-related behavior. Indeed, on the contrary, some scholars even suggest a loss of perceived autonomy resulting from self-monitoring technologies (13–17). As such, the literature on HSMAs and employee autonomy is inconclusive with several gaps addressed by this research.

First, employers providing HSMAs may impact the relative freedom employees experience in the use of such HSMAs and the self-regulation of their health-related behavior. At first sight, the provision of HSMAs might suggest honorable intentions. Counter-effects however might emerge that affect employees' sense of autonomy in self-regulating their health-related behavior. The use of worksite HSMAs makes the norms and standards for healthy behavior that are usually latent yet imposed by external entities (e.g., health agencies, employers) salient (18, 19). SDT suggests that if this happens, employees may feel that the locus of control over their health-related behavior shifts from internal to external. This potentially decreases their perceived autonomy. Therefore, our first research goal is to investigate the effects of employer-provided HSMAs on employees' perceptions of autonomy regarding the self-regulation of health-related behavior.

Second, HSMAs provide users with feedback information on specific aspects of their bodily functioning and health-related behavior. This information is assumed to facilitate the autonomous self-regulation of healthier behavior. This feedback usually focuses on discrepancies between one's actual health-related behaviors and standards set for those behaviors, which can be termed as “performance feedback” (20). However, one form of feedback that has hardly been used and examined in the HSMA context is “developmental feedback.” Developmental feedback includes information that facilitates recipients to learn, develop, and make adaptive behavioral changes (20). SDT suggests that developmental feedback may boost autonomy and intrinsic motivation for learning and improvement, whereas the evaluative and controlling information provided by performance feedback may inhibit feelings of autonomy (9). Therefore, our second research goal is to investigate the potentially moderating role of feedback focus (performance vs. developmental) in HSMAs' effects on perceived autonomy.

Third, individual differences, such as initial health condition may influence how employees respond to HSMAs in terms of perceived autonomy in self-regulating their behavior. Previous research showed that employees with poorer self-rated health respond more negatively to health checks with feedback than do healthier respondents (3). Less healthy employees reported experiencing less control over their health-related behavior and feared that health measures imposed by their employer would invade their privacy and interfere with their sense of personal autonomy (3). Therefore, our third research goal is to examine whether an employee's state of health influences HSMAs' effects on perceived autonomy.

Fourth, health metrics provided by HSMAs such as activity trackers capture daily activities that are carried out both within and beyond the workplace. Further, the standards set for physical activity (e.g., 10,000 steps a day) are usually not limited to the workplace. They are flexible standards for self-regulation of employees' health-related behavior during both work and private time. Although HSMAs thus appear to blur the lines between work and private time, employees may establish different autonomy feelings in the self-regulation of their health-related behavior in the workplace and at home. Employees may feel that HSMAs provided by their employer invade their private time and thus especially interfere with their sense of autonomy at home. Hence, to address these potentially different autonomy effects of HSMAs across work and private domains, we include measures of both work health autonomy and home health autonomy. Thus, our fourth research goal is to explore whether the effects of HSMAs that focus of feedback and health status are different for employees' perceptions of health autonomy at work and at home.

This study contributes to the HSMA research literature by using insights from SDT and feedback literature to examine the basic assumption underlying the use of HSMAs: that their self-management function promotes employees' perceptions of autonomy in self-regulating their health-related behavior. Our research shows that the type of feedback (performance vs. developmental) that employees obtain from HSMAs, in conjunction with their health condition, affects their perceived autonomy. Also, the effects of feedback and health condition on health autonomy perceptions are different at work and at home. These findings lead to guidelines for the effective use of HSMAs in different settings (work and at home) and for employees with different health conditions.

## Theory and Hypotheses Development

An overview of relevant findings from previous studies is provided here, leading to the development of three hypotheses about the effects of HSMAs on perceived autonomy, and how feedback focus and health moderate these effects. We then argue that autonomy should be considered both at work and in private time, leading to an explorative question about the effects of HSMAs for both work health autonomy and home health autonomy.

### HSMAs and Perceived Autonomy in the Self-Regulation of Health-Related Behavior

In the present research, we focus on the use of HSMAs, specifically the Fitbit One activity tracker. HSMAs provide users with feedback information on bodily functioning and health-relevant behaviors such as heart rate, steps taken, stairs climbed, and intensity of physical activity. Such devices are used in various domains, ranging from clinical settings for disease management (18) to occupational settings for disease prevention and health promotion (2, 6).

Reviews evaluating the effectiveness of different methods for promoting physical activity reveal that activity trackers can be very effective in increasing the number of steps participants take (6, 21). This increase in activity however does not by definition imply an increase in perceived autonomy of users. On the contrary, Owens and Cribb (19) argue that HSMAs do not inherently increase autonomy, and are even likely to decrease it, because externally imposed norms and values are likely to undermine genuinely autonomous deliberation by users. To date, research has not systematically and empirically examined how HSMAs influence employees' perceived autonomy in self-regulating their health-related behavior. Therefore, we aim to address this gap in the research literature.

SDT (9, 10) is seen as a promising framework for the study of autonomy in the self-regulation of health-related behavior. This theory contends that the quality of human motivation for regulating behavior varies along a continuum from autonomous motivation to externally controlled motivation. Individuals are autonomously motivated if they experience an internal locus of causality and self-determination in the self-regulation of goal pursuits. In contrast, controlled motivation is present when individuals experience an external locus of causality in goal pursuits, which occurs when their goal-directed behavior is controlled and regulated by externally imposed norms, standards, or sanctions. Research has shown that an increase in perceived autonomy promotes effective cognitive, affective, and behavioral self-regulation of health-related behavior (11, 22–26).

The first goal of this study is to examine the effect of a workplace HSMA intervention on employees' perceptions of autonomy in self-regulating their health-related behavior. Specifically, using an experimental field study in a company in the healthcare industry, we examine whether the use of an activity tracker (Fitbit One) provided by the employer increases or decreases the sense of autonomy that employees experience in regulating their health-related behavior. Here, we build two competing hypotheses regarding the effects of HSMAs on autonomy.

Using HSMAs enables employees to self-monitor their personal fitness metrics, and to become aware of the extent of their physical activity. This self-awareness facilitates users to reflect on their personal health situation and then to focus on goal setting, action planning, and actual engagement in physical activities to improve their health (21). This reliance on self-regulation makes employees responsible for their own health and enables them to independently self-manage their health-related behavior. SDT argues that self-responsibility and self-direction facilitate a more self-determined form of motivational regulation of behavior (27). Therefore, the first part of our competing hypothesis predicts that *HSMAs have a positive effect on employees' perceptions of autonomy in self-regulating their health-related behavior* (Hypothesis 1a).

However, even though HSMAs aim to facilitate autonomy in self-regulating health-related behavior, HSMAs might also interfere with the development of autonomous self-regulation. First, employer-provided HSMAs have been found not to be value-free (17), and may impose norms and standards, or expectations, for health-related behaviors. Specifically, by expecting employees to use HSMAs such as activity trackers, employers not only highlight health values but also impose guidelines, norms, or standards for physical activity (e.g., 10,000 steps a day), even if these are not explicit. As a result, employees may feel that the HSMAs interfere with their personal autonomy and free choice to behave in ways that the employer sees as undesirable, unfit, and unhealthy (18). They may perceive the use of HSMAs as a form of surveillance and control, leaving them no real choice, even if the employee is the only person with access to the data.

Second, HSMAs, such as activity trackers, focus on self-regulating health-related behaviors not only in the workplace but also in private life. For example, goals set for physical activity (such as 10,000 steps a day) are formulated as fluid goals that transgress and blur the border between work and private spheres (16, 28). With this continuous exposure to HSMAs, both in work and in private time, employees may experience the HSMAs as invading their privacy and decreasing their personal autonomy (16). Accordingly, based on these two arguments that HSMAs may constrain free-choice behavior and interfere with privacy, the second part of our competing hypothesis argues that *HSMAs have a negative effect on employees' perceptions of autonomy in self-regulating their health-related behavior* (Hypothesis 1b).

### The Moderating Role of Focus of Feedback

The essence of HSMAs is to provide feedback information on health-related behavior so that users can adjust their behavior to meet desired standards. HSMAs usually deliver performance-oriented feedback, which can be defined as information concerning discrepancies between one's actual performance (e.g., 6000 steps per day) and the performance standard (e.g., 10,000 steps per day) (29). Such information focuses on past performance, while its valence is critical in determining one's current and future behavior in regulating progress toward a standard (20). Another type of feedback is developmental feedback, defined as helpful or valuable information that enables the recipient to learn, develop, and make improvements (30). As such, this type of feedback focuses on the future rather than the past, with the feedback providing the recipient with developmental information that is helpful in improving certain performance dimensions (20).

We offer two arguments for why focus of feedback could moderate the effects of HSMAs on autonomy. First, using only performance feedback may tend to increase the salience of the potentially inhibitory effects of HSMAs on autonomy. This is because performance feedback highlights norms and standards for healthy behavior that are construed and imposed by external entities (i.e., employer or health agencies) rather than freely determined by the feedback recipients themselves (29). Due to this external imposition of health norms and standards, employees may perceive performance feedback as evaluative and controlling information intended to subtly force them to adapt their health-related behavior in line with the externally imposed standards. Consequently, HSMAs that only use performance feedback are likely to induce an external rather than an internal locus of causality in employees for regulating their health-related behavior.

Second, in contrast, the use of developmental feedback may tend to boost the salience of the potentially supportive effects of HSMAs on autonomy. This is because developmental feedback is informational in nature and fosters an orientation toward learning and development (20). Specifically, developmental feedback provides meaningful information that enables employees to learn why the recommended health-oriented behavior is important. Moreover, developmental feedback offers employees alternative options and ways to achieve behavioral change and health improvements. Since these options provide choice and self-direction, developmental feedback enables employees to experience themselves as autonomous initiators and regulators of health promotion actions (11, 22). Accordingly, we hypothesize that *the focus of the feedback moderates the effects of HSMAs on employees' perceptions of autonomy in self-regulating their health-related behavior, such that the effects are more positive when employees receive developmental feedback in addition to mere performance feedback* (Hypothesis 2).

### The Moderating Role of Health

Employees differ in their health status, and these individual differences seem to influence how they respond to workplace health promotion programs. Recent research shows that less healthy employees experience more difficulties in adhering to healthy lifestyle behaviors recommended by guidelines (31, 32). They feel that workplace health promotion programs invade their privacy and go against their personal autonomy (3). Given this finding, we examine how differences in individual health conditions moderate the effects of HSMAs on autonomy. Here, we use body mass index (BMI) as a holistic measure of health (33). We use BMI as a proxy of health because of its high predictive validity across many health outcomes and widespread use in population and medical research, and because it is a convenient and simple measure of health that can be self-reported by individuals without requiring inputs from medical authorities (33).

We discuss two reasons why BMI might moderate the effects of HSMAs on employees' perceptions of autonomy in self-regulating their health-related behavior. First, HSMAs may encourage weight-based stereotypes that overweight individuals are lazy and unattractive, and lack self-discipline and willpower, thus assigning responsibility and blame to overweight individuals with unhealthy lifestyles (32, 34). As a consequence, workplace health promotion measures may be seen as a violation of privacy and a painful interference with personal autonomy to live life on one's own terms (34). Moreover, employees with a high BMI may see the use of HSMAs as an attempt by their employer to subtly press them to take action to reduce their weight, thereby harming their sense of self-determination and autonomy. In contrast, as thinness is seen as the healthy ideal (33), employees with a healthy BMI will not feel stigmatized when an HSMA provides feedback information about suboptimal health-related behaviors. Not feeling stigmatized, and helped by the feedback from the HSMA, they are more prepared, than high BMI employees, to reduce the suboptimal behaviors identified and stay healthy.

Second, employees with high BMI often need to make more drastic lifestyle changes than employees with healthy BMI to meet the standards for healthy physical activity and weight that are made salient by HSMAs. Such changes are far more difficult to achieve for overweight individuals (31), leaving them with a much greater likelihood of failing to adhere to the recommended guidelines (32). Failure adds to the stigmatization and stereotyping of overweight individuals, increasing their vulnerability to psychological distress and the risk of backsliding into unhealthy lifestyle behaviors (32). Consequently, employees with high BMIs may feel they are less able to regulate and change their lifestyle behaviors to meet the HSMA standards and recommended guidelines. This decreases their sense of autonomy and self-regulation. In contrast, healthy employees with an optimal BMI often need to make far less difficult lifestyle changes to meet the recommended guidelines and standards. As such, their healthy BMI facilitates self-efficacy and self-control in regulating health-related behavior, which reinforces perceptions of self-direction and autonomy. Based on the above reasoning, we hypothesize that *BMI moderates the effects of HSMAs on employees' perceptions of autonomy in self-regulating their health-related behavior, such that the effects are more strongly negative (or less strongly positive) for employees with higher BMIs than for employees with lower BMIs* (Hypothesis 3).

### Health Autonomy at Work and at Home

HSMAs such as activity trackers provide users with physical activity metrics that are usually measured on a daily basis and capture activities carried out within and beyond the workplace. Further, the standards set for physical activity (e.g., 10,000 steps a day) are not specified exclusively for the workplace but are fluid goals for health-relevant behaviors in both work and private lives. Thus, besides their influence on autonomy and control of health-related behavior in the workplace, HSMAs may also affect the sense of autonomy that employees experience in regulating their health-related behavior at home. On the one hand, the fluidity of HSMAs may enhance perceived autonomy in both domains. The pursuit of health-related goals (e.g., 30 min of moderate intensity exercise each day) is not limited to the work domain but continues into private time. This fluidity in goal pursuits in work and private domains is comparable with tele-working that may facilitate flexibility to reach both work and family goals in the same time frame (35). However, on the other hand, employees may experience the continuous exposure to the HSMA's demands as an interference with their self-determination in personal life. This might decrease their perceived autonomy in self-regulating their health-related behavior. Accordingly, we examine the potentially different effects of HSMAs on perceived autonomy at work and at home. We do so by including measures of both Work Health Autonomy (WHA), defined as perceived autonomy to regulate health-related behavior during working hours, and Home Health Autonomy (HHA), referring to perceived autonomy to regulate health-related behavior during private time. Previous research on autonomy in the workplace does not lend itself to deriving theoretical argumentation for different HSMA effects on these two distinct types of health autonomy. Therefore, the distinct measures of work and home health autonomy are studied in an exploratory fashion, rather than attempting to develop and test theory-driven hypotheses. Thus, our exploratory research question is whether HSMAs, feedback focus, and BMI have different effects on employees' perceptions of work health autonomy and home health autonomy.

## Methods

### Design, Sample, and Procedure

To examine the effects of employer-provided HSMAs on employees' perception of autonomy in the self-regulation of their health-related behavior, we executed a pretest-posttest randomized two-phase field experiment study in a company in the Netherlands. The study included a 4-weeks HSMA intervention with a feedback factor (performance vs. development feedback) and pretest (T1) and posttest (T2) measurements of participants' perceptions of autonomy. After the experiment period, a series of interviews was conducted with employees with varying BMIs.

#### Setting

The company involved is a medium-sized hospital that had started an organization-wide workplace health promotion program to facilitate the health, well-being, and work-life balance of its employees. The company employs a variety of workers such as nursing and technical staff, specialists, and support staff, and office workers with varying levels of mental and physical activities. As one-size-fits-all advices for health promotion may not match such a heterogenous workforce, the hospital management team decided to provide employees with measures through which employees could self-regulate their own unique health behavior including an activity tracker (Fitbit One). However, before implementing this activity tracker throughout the hospital, the management team wanted to investigate its effects and asked us to conduct an experimental field study. The experimental protocol for the study was approved by the designated research ethics committee and sent to the ethics committee of the healthcare institute for information purposes.

#### Participants

Participants were recruited by sending e-mails and a newsletter to all employees in which they were informed about the experiment and offered the opportunity to participate. Employees who were interested in the use of HSMAs are likely to be overrepresented in the sample. However, given that workplace health promotion programs usually rely on voluntarily participation and that participation rates vary from 10 to 64% (with an average of 33%) (36), we think that the sample in the present experimental field study is representative for the total population of employees that voluntary participate in health promotion programs. In total, 166 employees responded out of 1,525 potential participants (11%). Of these, two were unable to participate due to lengthy absences during the experiment period. Of the remaining 164 employees, 30 were assigned to a pilot group that was used to test and improve the methodological, technical, and logistical features of our experiment. Eleven participants were interviewed after finishing the experiment. All participants in both the pilot group and the main experiment gave an informed consent.

#### Pilot

During the pilot, the technical feasibilities of the HSMAs and data-logging system were tested and evaluated, and modifications were made where necessary. Moreover, small alterations were made to improve the wording of some questionnaire items, and additional information was added to the information sheet for new participants, especially about the use of participants' research accounts for data gathering and preventing them from linking the HSMA to their own smartphone.

#### Main Experiment

**Figure 1:**
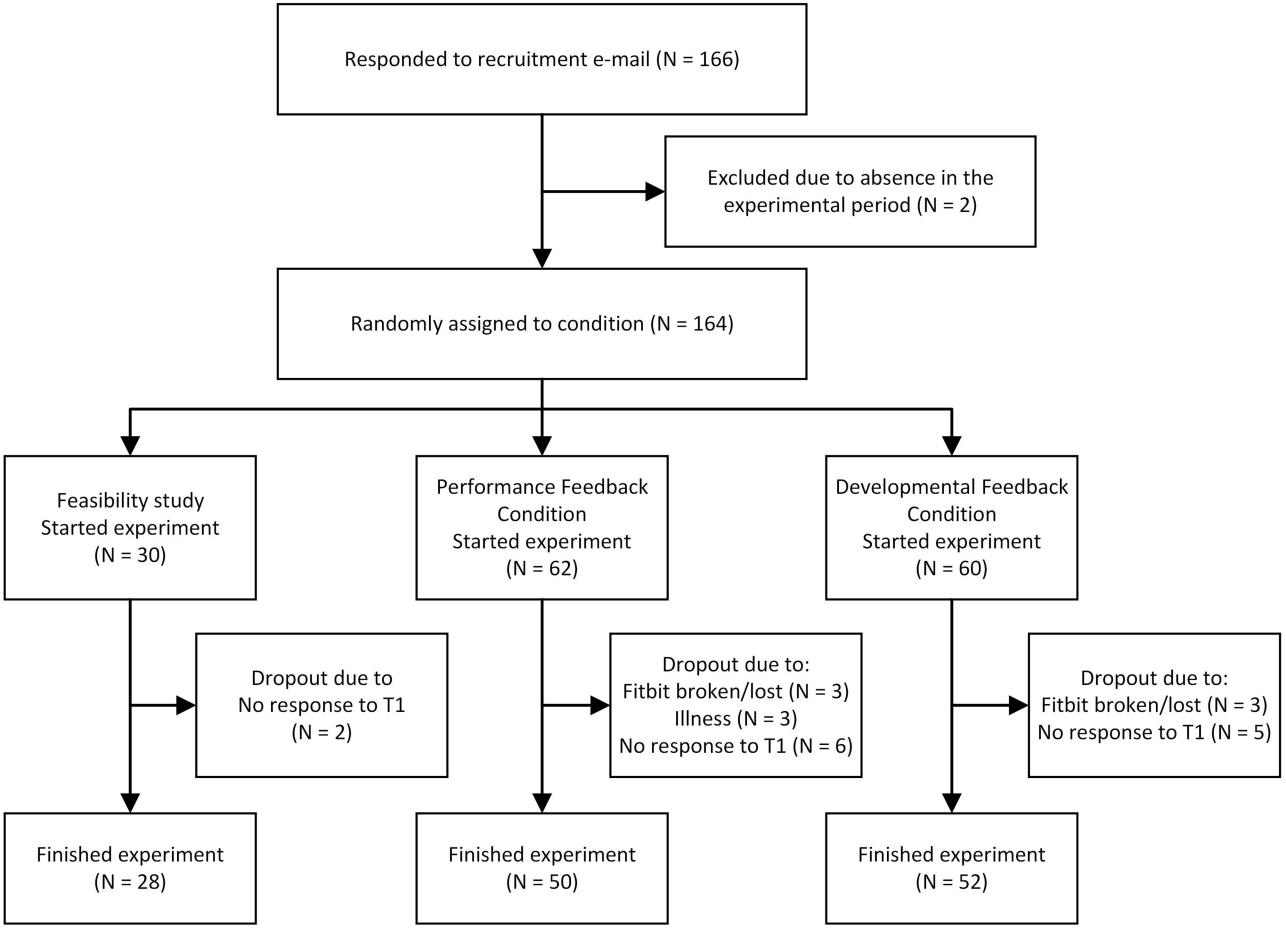
Participation flow chart.

The 134 participants that were not involved in the pilot were randomly assigned to either the performance feedback condition (PFC; *N* = 68) or the developmental feedback condition (DFC; *N* = 66). These 134 participants were invited by email to complete an online questionnaire at the pretest measurement point, and 122 completed the questionnaire (N_PFC_ = 62, N_DFC_ = 60). The 122 participants that completed this pretest were provided with an HSMA. Of these 122, 20 dropped out, either because they did not use their HSMA or because they did not complete the post-experiment questionnaire distributed after the 4-weeks intervention period (see Figure 1 for detailed participant flow chart). Consequently, the final sample included 102 participants (N_PFC_ = 50, N_DFC_ = 52). The retention rate of the participants therefore is 76.1%, which is higher than most e-health interventions in the workplace showing high to very high attrition rates (37), with only 20% of studies reaching a retention rate of 75% or more (38). Of the remaining participants, 84% were female. The participants average age was 46 (*SD*_age_ = 10), and their average employment duration was 11.9 years (*SD*_employment_ = 10.4). Most participants (64%) had a higher education or university degree, while 25% had a vocational degree, and 11% had less formal education. The spread of employees across the job spectrum was considered satisfactory, including both administrative and medical personnel, ranging from management and medical specialists to nursing, administrative, and technical staff.

### HSMA Intervention and Manipulation of Feedback Focus

#### Procedure

After completing the pre-test questionnaire, the participants were informed about the HSMA intervention following a standardized procedure. This involved a letter stating the goal of the study, the duration of the experiment (4 weeks), the expectations of the participants (to wear a Fitbit for the 4 weeks, complete a post-test questionnaire, and participate in a focus group or interview if asked to), the expected time-investment, and information on data confidentiality. Participants were not expected to use any smartphone or other applications connected to the device, and all data were collected and stored in accounts used only for research purposes. All participants were made aware that their employer did not have access to the data obtained using the activity tracker. The participants then received an activity tracker that measured their number of steps taken, stairs climbed, and minutes of light, moderate, and heavy activities during the day.

#### Manipulation of Feedback Focus

The screen of the activity tracker provided the participants with their personal activity metrics on a daily basis. In addition, they received an email once a week reporting their physical activity metrics in which the focus of the feedback was manipulated. Specifically, participants under the *performance feedback* condition received only performance feedback information showing factual metrics as assessed by the activity tracker for each of the past 7 days (e.g., October 18: 8,000 steps, 14 stairs, 77 min light activity, 20 min moderate activity, and an estimated calorie use of say 2,200 kCal) and the general norms for these measures (10,000 steps a day and a calorie intake of 2,000 kCal for women, 2,500 kCal for men). Participants under the *developmental feedback* condition in addition received development feedback, giving advice on how work-related activities could be altered in order to encourage a healthy behavior pattern and lifestyle (see Appendix 1 for feedback examples). These developmental feedback mails included information on the intensity of daily activities, ways to increase their daily activity, tips and tricks to adjust and sustain exercise patterns, and information on food and nutrition. This feedback was based on advice from the Netherlands Nutrition Center, the National Institute of Public Health and the Environment, and the Knowledge Center for Sport and Physical Activity. The developmental feedback information in the e-mails was refreshed weekly, and built upon the information given in the previous week(s).

### Measures

#### Autonomy

We adapted the three items of the Autonomy scale of the Job Diagnostic Survey (39) developed by Hackman and Oldham (40) to assess participants' perceptions of work health autonomy (WHA) and home health autonomy (HHA). We pretested the suitability of the individual items of this adapted autonomy scale and solved small wording issues that led to confusion with some of the participants. For WHA, one item from the initial Autonomy scale was applied to capture autonomy experiences for both the work as a whole and individual tasks, resulting in four items for WHA. Two example items are “I can independently decide how to take my health into account when executing my job” (WHA) and “In my private time, I'm free to decide whether I want to do something about my health and health-related behavior” (HHA). We used a five-point Likert response scale ranging from 1 (strongly disagree) to 5 (strongly agree). See Table 1 for items and statistics of an exploratory factor analysis testing the discriminant validity of the two autonomy scales.

**Table 1:** Results of factor analysis for WHA and HHA.

**Items**	**WHA**	**HHA**
**Work Health Autonomy**		
In my work, I have the opportunity to plan my work activities such that they will benefit my health	**0,869**	−0,067
I can independently decide how I want to take my health into account in the execution of my work	**0,860**	−0,069
I can decide how to execute individual work tasks in the most healthy way	**0,843**	0,063
In my workplace, I have the freedom to take initiatives that benefit my health	**0,840**	0,076
**Home Health Autonomy**		
In my private time (outside of work), I feel totally free to decide whether or not I want to do something about exercise and health	0,094	**0,701**
I feel pressured by my employer to include exercise and health in the planning of my private activities (*R*)	−0,109	**0,854**
My employer restricts me in my freedom regarding how I deal with exercise and health in my private time (*R*)	0,002	**0,869**
Eigenvalues	2,939	1,986
Percentage explained variance	41,98	28,37
Cronbach's alpha	0.871	0.730

*(R) Indicates a reverse-worded item*.

*Bold values indicate highest factor*.

#### BMI

Participants reported their body weight and height. These self-reported values were used to calculate their Body Mass Index.

#### Control Variables

We included the demographic variables of gender, age, organizational tenure, education, and previous experience with activity trackers (yes vs. no) as control variables as these variables could potentially influence participants' perceptions of work and home health autonomy.

### Statistical Analyses

To examine the impact of the HSMA intervention (activity tracker) on perceptions of autonomy in self-regulating health-related behavior during work and personal time, paired-sample *t*-tests were conducted to test differences between pretest (T1) and posttest (T2) autonomy (Hypotheses 1a and 1b). This was done for WHA and HHA separately to investigate our explorative question. Having formulated competing hypotheses on the direction of the autonomy effects of HSMA, we used two-tailed tests using a significance level of 0.05. Further, multiple regression analyses were conducted to test the hypothesized effects of feedback focus and BMI on T2 autonomy in self-regulation of health-related behavior, thereby including T1 autonomy as a covariate (Hypotheses 2 and 3). Specifically, the regression analyses consisted of two steps. The first step, in addition to the covariate of T1 autonomy, included dummies for feedback focus (performance = 0, developmental = 1) and BMI to test their effects on T2 autonomy. The second step included the cross-product term of feedback focus and BMI to explore their possible interaction effects on T2 autonomy. Our hypotheses had specified the direction of the moderating impacts of feedback focus and BMI on the autonomy effects of HSMA. Therefore, we used one-tailed tests with a significance level of 0.05. To facilitate interpretation and minimize multi-collinearity problems when testing interaction effects, we used cross-product terms of standardized predictors. Again, we ran separate regression analyses for work (WHA) and home health autonomy (HHA) to examine our explorative question.

### Second Stage of the Study: Interviews

To explore the mechanisms underlying the moderating effects of feedback and BMI that we identified (see Results section), additional qualitative data were gathered after completing the experimental period. The first author conducted interviews with 11 participants who were spread across the BMI spectrum. Two participants had BMI values lower than 20, two had BMI values between 20 and 25, three had BMI values between 25 and 30, two had BMI values between 30 and 35, and two had BMI values above 35. Interview requests were sent randomly to four participants in each BMI-category, and upon positive response an interview was scheduled. Seven interviewees were in the performance feedback condition, four interviewees were in the developmental feedback condition. The interviews were semi-structured, and protocol questions were focused on how interviewees had experienced and responded to the HSMA feedback in regulating their health-related behavior in the workplace and in private time. The duration of the interviews was 25–45 min, and all the interviews were conducted during or immediately after working hours, unless the interviewee requested otherwise. All interviews were taped and transcribed, and a common codebook of 35 codes was generated by having two authors separately and iteratively code one interview, and then compare and align their codes. This codebook was validated by analyzing two further interviews that were coded using this codebook by both these authors, resulting in an interrater reliability (Holsti's coefficient) of 0.78 (41). After this validation check, the codebook was used by the first author to code all 11 interviews. Following the coding of the interviews, network diagrams of co-occurring and consecutive codes were made for each interview separately and checked for consistency in interpretation by another author. The individual diagrams were clustered into sub-groups based on BMI score and feedback type to trace any patterns within and between sub-groups of interviewees. This allowed us to further analyze and clarify the roles of both BMI and feedback focus in the autonomy effects of HSMAs.

## Results

### Exploratory Factor Analyses

In order to get some evidence for the discriminant validity of the autonomy scales that were created by adapting the Autonomy scale of the Job Diagnostic Survey, the items of the WHA (four items) and HHA (three items) scales were factor analyzed using principal components extraction and oblique rotation. As can be seen in Table 1, two factors emerged with eigenvalues >1, accounting for 70.35 percent of the variance. Each item “loaded” on its appropriate factor, with primary loadings exceeding 0.701 and cross-loadings lower than 0.094. The correlation between the two factors was insignificant.

### Equivalence of Experimental Feedback Groups

Prior to hypothesis testing, we conducted a one-way analysis of variance (ANOVA) to check the pretest equivalence of the variables across the two experimental feedback groups. That is, we tested whether the participants in the performance feedback group systematically differed from the participants in the developmental performance group with respect to their scores on the demographics of gender, age, organizational tenure, experience with HSMAs, education level, and BMI, and on the study variables of work health autonomy and home health autonomy at the pretest measurement point (T1). As can be seen in Table 2, the ANOVA results did not indicate significant differences for any of the variables, showing pretest equivalence of the variables across the two feedback groups.

**Table 2:** ANOVA results.

	**Sum of squares**	**df**	**Mean square**	***F***	**Sig**.
Home health autonomy pre-test	0.007	1	0.007	0.019	0.890
Work health autonomy pre-test	0.109	1	0.109	0.127	0.722
HSMA experience	0.094	1	0.094	0.374	0.542
Year of birth	4.588	1	4.588	0.041	0.839
Education level	0.189	1	0.189	0.164	0.686
BMI	23.313	1	23.313	1.904	0.171
Tenure	54.932	1	54.932	0.502	0.480
Gender	0.028	1	0.028	0.207	0.650

### Descriptive Statistics

Table 3 presents means, standard deviations, and correlations for all the variables included. The correlations indicate that none of the control variables are significantly related to the autonomy variables, leading us to exclude them from our analyses to avoid biased parameter estimates (42).

**Table 3:** Means, standard deviations, and zero-order Pearson correlations for variables (*N* = 102).

	**Mean**	**SD**	**HHA pre-test**	**HHA post-test**	**WHA pre-test**	**HHA post-test**	**Feedback type**	**BMI**	**HSMA experience**	**Type of job**	**Year of birth**	**Education level**	**Tenure**
HHA pre-test	4.62	0.61	1										
HHA post-test	4.34	0.79	0.351^**^	1									
WHA pre-test	3.44	0.92	0.031	−0.013	1								
WHA post-test	3.54	0.90	0.019	0.104	0.635^**^	1							
Feedback type^1^	0.51	0.50	−0.014	0.009	−0.036	0.073	1						
BMI	24.48	3.51	−0.277^**^	−0.287^**^	0.060	−0.082	0.137	1					
HSMA experience^2^	0.45	0.50	0.119	−0.033	0.054	−0.058	0.061	0.153	1				
Type of job^3^	0.56	0.50	−0.118	−0.199	−0.081	0.037	0.094	0.037	0.042	1			
Year of birth	1971.55	10.46	−0.159	0.012	0.038	−0.053	−0.021	−0.014	0.123	0.284^**^	1		
Education level^4^	4.70	1.07	0.039	0.093	0.072	−0.026	−0.040	−0.072	0.166	−0.019	0.202	1	
Tenure	11.88	10.43	−0.075	−0.031	−0.096	−0.002	0.071	0.034	−0.123	−0.078	−0.558^**^	−0.429^**^	1

* *Correlation is significant at the 0.05 level (2-tailed)*.

** *Correlation is significant at the 0.01 level (2-tailed)*.

1 *Zero is performance feedback, one is development feedback*.

2 *Zero is no previous experience, one means participant has used/uses an HSMA*.

3 *Zero is mainly office work, one is physically active work*.

4 *Range is 0–6, 0-1 reflects low education level, six is a university degree*.

### Hypothesis Testing

#### Pretest-Posttest Differences in Autonomy

**Figure 2:**
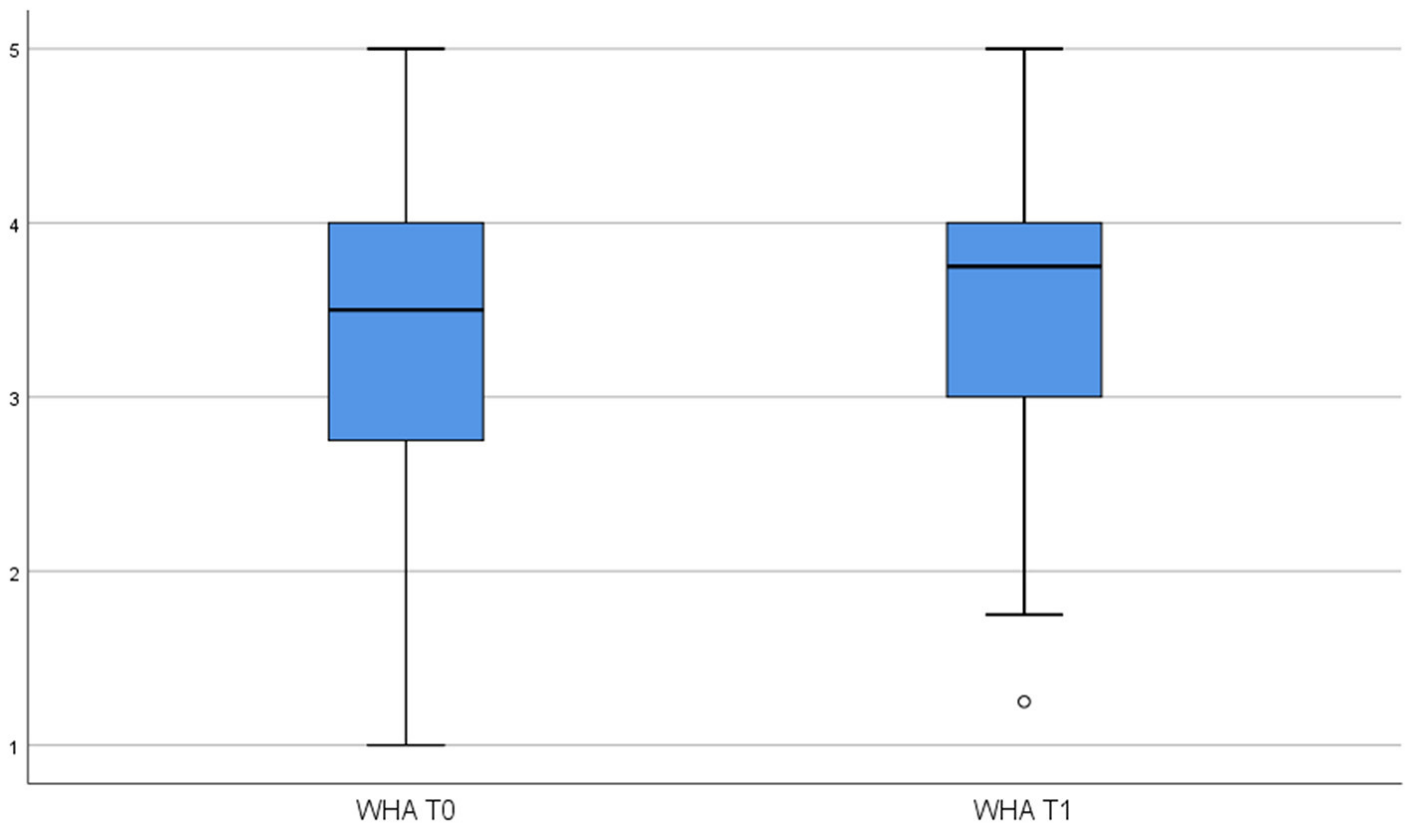
Results of paired sample *t*-tests WHA.

**Figure 3:**
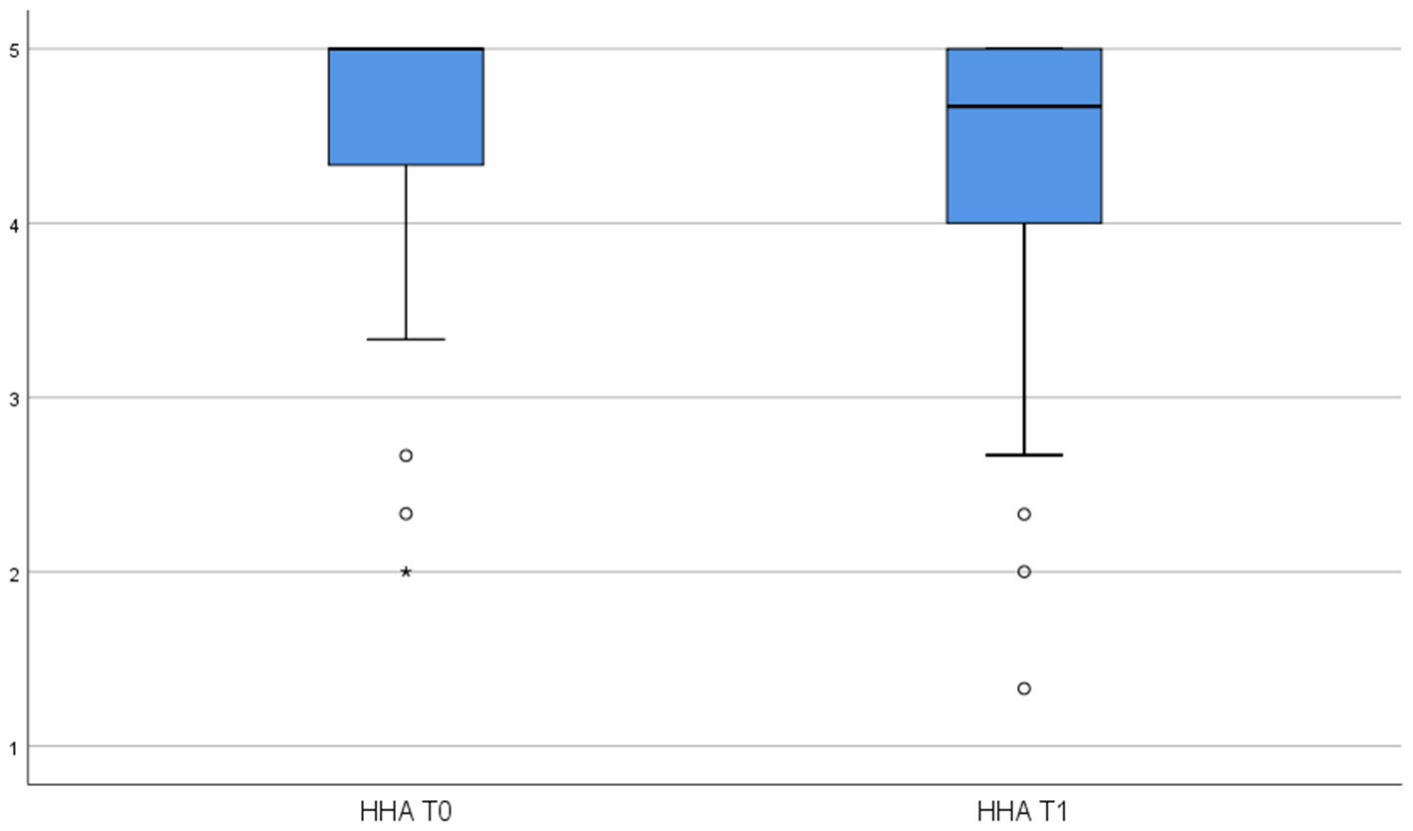
Results of paired sample t-tests HHA.

To test Hypothesis 1, we examined whether the use of the HSMA activity tracker influenced employees' perceptions of WHA and HHA. Specifically, we conducted paired-sample *t*-tests to determine if there were significant differences between pretest and posttest means for the respective autonomy variables. Table 3 reports the pretest-posttest means, standard deviations, and *t*-values for both WHA and HHA. These are visualized in Figures 2, 3. The difference between the pretest and posttest means is not statistically significant for WHA, whereas it is significant for HHA (*t* = −3.184, *p* < 0.01) indicating that the use of HSMAs decreased employees' perceptions of autonomy in regulating their health-related behavior in their private time. Thus, based on these results, Hypothesis 1a, predicting a positive effect of HSMAs on employees' perceptions of autonomy in self-regulating their health-related behavior, was rejected, whereas Hypothesis 1b, predicting a negative effect of HSMAs on perceived autonomy, was confirmed for HHA but not for WHA.

#### Effects of Feedback Focus and BMI

Regression analyses, separately conducted for WHA and HHA at T2, showed that the feedback focus (performance vs. developmental) had a marginally significant and positive effect on T2 WHA (*b* = 0.10, *t* = 1.44, *p* < 0.10, one-tailed test). In line with Hypothesis 2, this finding indicates that the effect of HSMAs on WHA was more strongly positive when employees received developmental feedback than when they received only performance feedback. Feedback focus had no significant effect on T2 HHA (*b* = 0.03, *t* = 0.44, *p* > 0.05, one-tailed test), which contradicts Hypothesis 2. Table 4 reports these regression results under Model 1.

**Table 4:** Results of paired-sample t tests.

	**Pretest**		**Posttest**		***t***	**df**	***p***
	**Mean**	***SD***	**Mean**	***SD***			
Work health autonomy	3.43	0.93	3.53	0.90	1.226	97	0.223
Home health autonomy	4.61	0.61	4.35	0.79	−3.184	98	0.002

Furthermore, as can be seen in Table 5 under Model 1, BMI had significant negative effects on both T2 WHA (*b* = −0.12, *t* = −1.73, *p* < 0.05, one-tailed test) and T2 HHA (b = −0.17, *t* = −2.16, *p* < 0.05, one-tailed test). These results indicate that the effects of the HSMAs on both WHA and HHA were more strongly negative for employees with high BMI levels than for employees with low BMI levels, a finding fully in line with Hypothesis 3.

**Table 5:** Regression results for work health autonomy and home health autonomy.

	**T2 work health autonomy**				**T2 home health autonomy**			
	**Model 1**		**Model 2**		**Model 1**		**Model 2**	
Predictor	b	t	b	t	b	t	b	T
Constant	3.54	51.77^***^	3.52	51.57^***^	4.34	59.99^***^	4.37	62.21^***^
Autonomy pretest	0.57	8.22^***^	0.58	8.43^***^	0.23	2.98^**^	0.23	3.15^**^
Feedback	0.10	1.44^†^	0.10	1.44^†^	0.03	0.44	0.04	0.50
BMI	−0.12	−1.73^*^	−0.10	−1.49^†^	−0.17	−2.16^*^	−0.19	2.62^**^
Feedback * BMI			0.12	1.75^*^			−0.21	−3.00^**^
*R* ^2^	0.42		0.43		0.16		0.23	
Adjusted *R*^2^	0.40		0.41		0.13		0.20	
*F*	23.29^***^		18.61^***^		6.18^***^		7.26^***^	

† *p < 0.10,*

* *p < 0.05,*

** *p < 0.01,*

*** *p < 0.001, one-tailed tests*.

**Figure 4:**
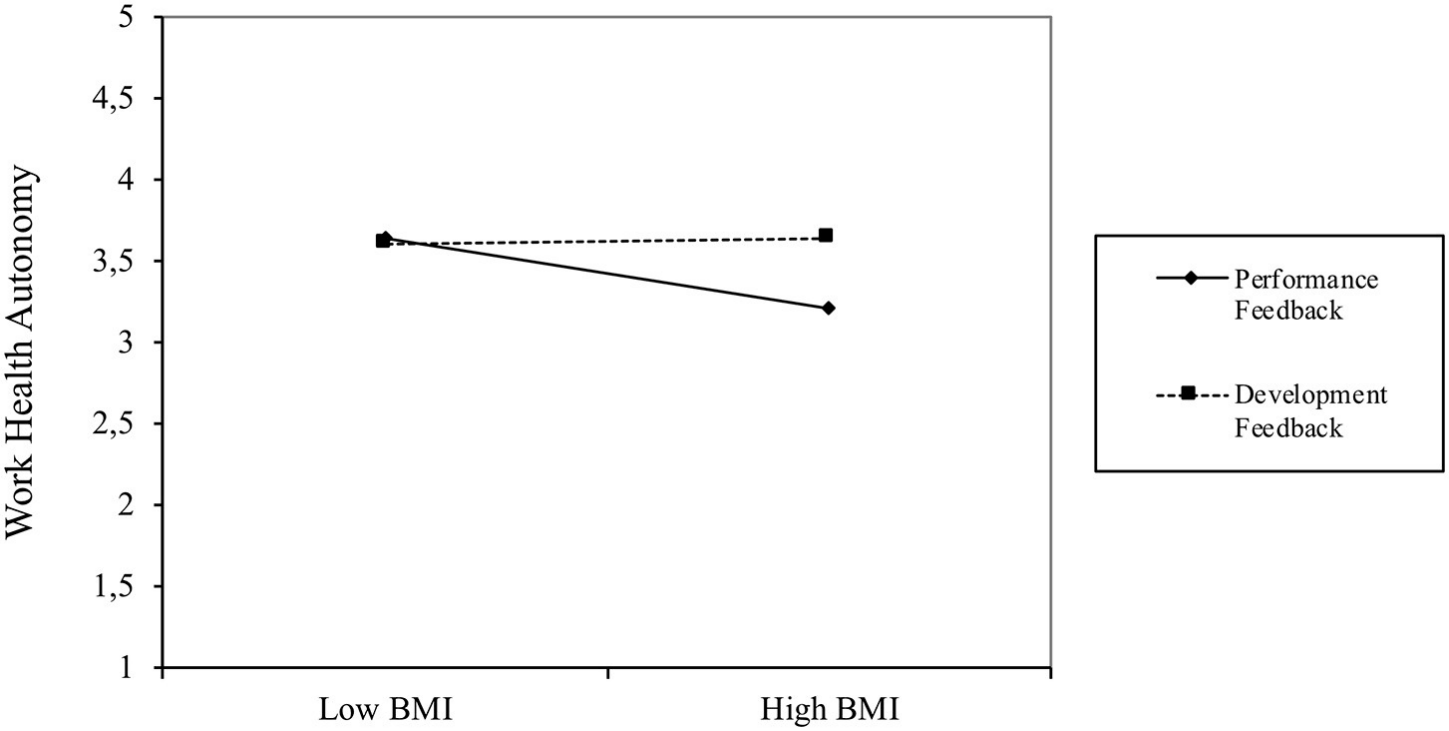
Pattern of interaction effect of BMI and feedback focus on T2 work health autonomy.

In addition, for exploratory reasons, we tested for interaction effects between feedback focus and BMI (see Table 4, Model 2). The interaction effect was significantly positive for WHA (*b* = 0.12, *t* = 1.75, *p* < 0.05, one-tailed test) and significantly negative for HHA (*b* = −0.21, *t* = −3.00, *p* < 0.01, one-tailed test). Additional simple slope tests (see Figure 4) indicate that BMI was significantly and negatively associated with T2 WHA (*b* = −0.23, *t* = −2.47, *p* < 0.05) for participants who had received only performance feedback, but that BMI was unrelated to T2 WHA (*b* = 0.02, *t* = 0.18, ns) for employees who had also received developmental feedback. Thus, the effects of the HSMAs on WHA were more strongly negative for employees with high BMI levels who received performance feedback, whereas BMI did not moderate the effects of HSMAs on WHA when employees received only developmental feedback.

**Figure 5:**
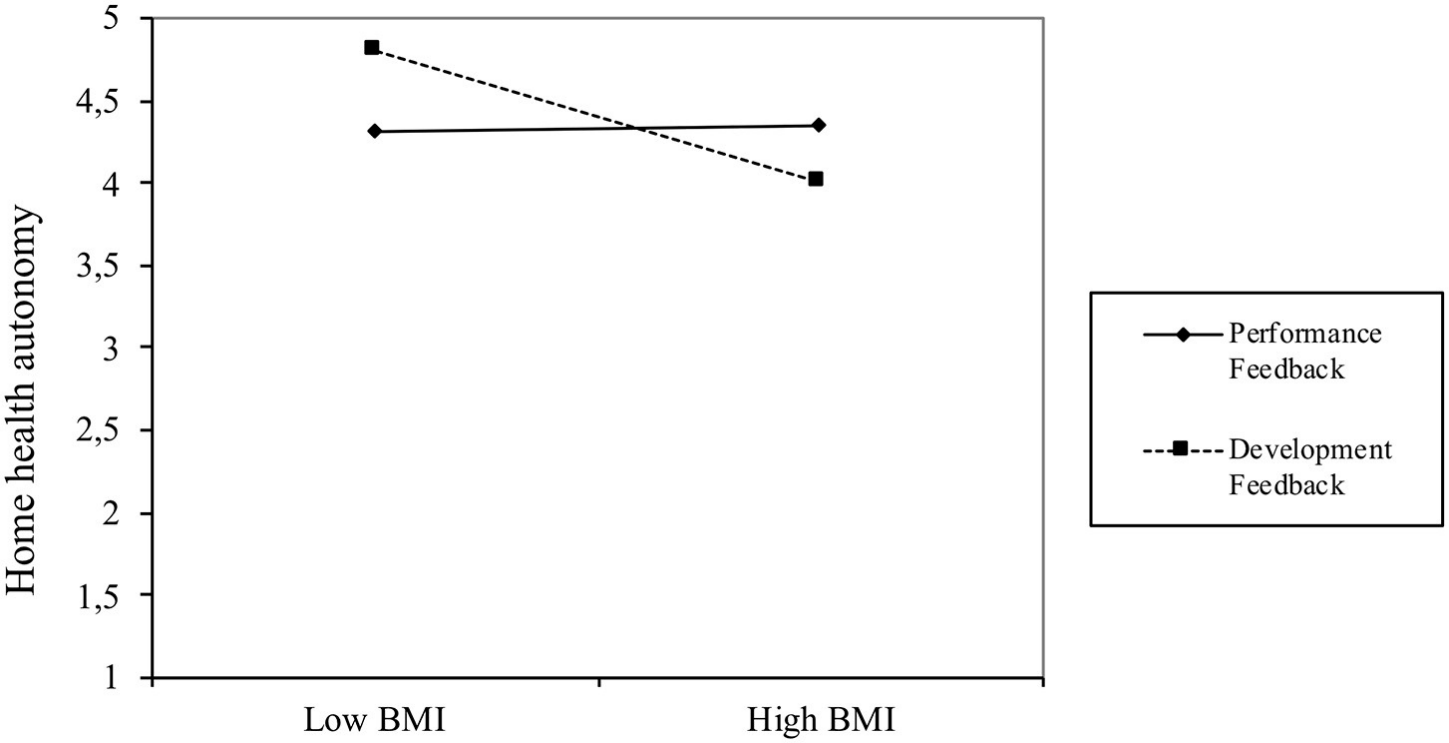
Pattern of interaction effect of BMI and feedback focus on T2 home health autonomy.

In contrast, the interaction plot displayed in Figure 5 shows that BMI was unrelated to T2 HHA (*b* = 0.02, *t* = 0.21, ns) for participants who received only performance feedback, whereas BMI was significantly and negatively related to T2 HHA (*b* = −0.41, *t* = −3.73, *p* < 0.001) for employees who received additional developmental feedback. As Figure 2 shows, with developmental feedback alone, the highest levels of HHA are to be found in low BMI employees, with the level of HHA decreasing strongly at higher BMI levels.

### Supplementary Analysis of Additional Qualitative Data

The qualitative interview research focused on understanding two of the main findings from the quantitative study:

1. Performance feedback group: the use of HSMAs resulted in a greater reduction in work health autonomy for employees with a higher BMI (see Figure 4)
2. Developmental feedback group: the use of HSMAs resulted in a greater reduction in home health autonomy for employees with a higher BMI (see Figure 5)

In order to identify the underlying mechanisms that cause these differences in perceptions of autonomy between employees with low and high BMIs, we asked the interviewees about their experienced autonomy both at work and at home, and the impact of the Fitbit and the received feedback on this autonomy. In this section, we present the effects that we uncovered and illustrate these with quotes from the interviewees.

#### BMI, Performance Feedback, and Work Health Autonomy

Employees with a high BMI experienced the standard norms highlighted in the performance feedback as very challenging and indicated that the use of the Fitbit made these norms more salient, whereas employees with a low BMI tended to interpret the performance feedback more loosely, and give it a positive spin:

*I discussed it with a colleague who also participated in the Fitbit experiment, and it really depends on what patient rooms you are assigned to. Some are at the front of the department, and then you have to walk a lot more compared to rooms close to the counter. […] And then I thought, I only make this number of steps, I really have to walk some extra kilometers. (Q1: Medical personnel, performance feedback, high BMI)*

*Yes, I often don't make the 10,000 steps, but that number is also something that was once made up. (Q2: Medical personnel, performance feedback, low BMI)*.

Further, employees with a high BMI commented that the performance feedback made them very aware of the fact that they could not achieve the 10,000 steps norm. They found this very confronting, leading them to express more negative emotions and feelings about the performance feedback they received. As such, high BMI employees seem to experience the performance feedback as more of a burden:

*Well, I thought I was quite active, and when I started [the experiment] I walked quite a lot […] But it was quite disappointing, how little you move or exercise at work. (Q3: Medical personnel, performance feedback, high BMI)*

*I now [after the experiment, AB] have an app that registers everything. […] and then I think, ooh, did I only walk so little? That is not a lot for a day like that! And then I get embarrassed about it, this isn't good, especially because I worked the entire day. (Q4: Administrative personnel, performance feedback, high BMI)*

Third, employees with a high BMI relatively more often experienced obstacles to self-regulating and intensifying activity in the work situation. That is, they tended to see more obstacles such as scheduling or work pressure issues. Moreover, employees with a high BMI felt less need to compensate for this lack of opportunity to self-regulate at work in the home situation:

*[…] No, because that is impossible. We don't have breaks, and no lunchbreak, so we pretty much work for 8 h straight. So, we can't go for a walk outside or something. (Q5: Administrative personnel, performance feedback, high BMI)*

*We discussed it [among colleagues], that it would be great to have the opportunity to go for a walk during lunch, but now we only have time to quickly finish eating and then our break is over. (Q6: Medical personnel, performance feedback, high BMI)*

*Because I have less spare time, I don't achieve it [the 10,000 steps]. And, as I said, sometimes [after work] I'm too tired, and then I start thinking that I would have to walk, no, I can't always make that. Time wise, or energy wise. (Q7: Medical personnel, performance feedback, high BMI)*

However, employees with a low BMI experienced more self-regulating options and less obstacles to move at work, and seemed to use the feedback from the HSMA to adapt their behavior in the work environment:

*I started taking the stairs. […] Otherwise I didn't really exercise more, but I took the stairs more often, because we're [at work] on the third floor and therefore climb three flights of stairs. (Q8: Medical personnel, performance feedback, low BMI)*

*Yes, I really think a thing like that [HSMA] helps to exercise more. Because I have sometimes caught myself thinking, darn, I'm taking the elevator [at work] when I should have taken the stairs, and I know I won't reach my step goal today. You are more conscious of what you do, and sometimes do things that you wouldn't have done otherwise. (Q9: Medical personnel, performance feedback, low BMI)*

Moreover, and in contrast to employees with high BMIs, employees with low BMIs related a low performance feedback score to their overall movement, both at work and at home. They expressed the view that a low performance score encouraged them to self-regulate and also move more in the home situation, especially when the work situation lacked opportunities to increase the movement pattern:

*Well, I was a bit lazy regarding exercising, and now I'm exercising at least once and often twice a week, really consciously. It is a bit dependent of my schedule, and you know, I'm taking the bike more often, and maybe taking longer walks with the dog to move more. (Q10: Medical personnel, performance feedback, low BMI)*

These differences in compensation behavior between the work and home environment are especially interesting because both employees with high and low BMIs mention that they do regularly exercise in their private time:

*I usually go to the gym 2 to 3 times a week, depending on my schedule. (Q11: medical personnel, performance feedback, high BMI)*

*I run, about once a week, and once a week I go for a spinning class, and in the weekend when the weather is ok I'm cycling a lot. (Q12: Administrative personnel, performance feedback, medium BMI)*

*Well, we have a dog, so I walk multiple times a day. And I do Pilates, which is good for my body strength, but I can't really see it in my Fitbit (Q13: Medical personnel, performance feedback, low BMI)*

Even though their general exercise levels outside of work are comparable, the reasons to alter the amount of exercise are different.

#### BMI, Developmental Feedback, and Home Health Autonomy

In this section, we focus on employees with high BMIs who received developmental feedback, and we aim to shed light on why their perceived autonomy to self-regulate their health in their private time declined, while it remained stable in working hours.

First, employees with both high and low BMIs that received developmental feedback reported becoming aware of more opportunities to self-regulate their health-related behavior in the workplace:

*Yes, well, due to that Fitbit, I no longer go to the restaurant to have lunch or dinner, just to not be tempted anymore regarding food. (Q14: Administrative personnel, development feedback, high BMI)*

*Yes, with that Fitbit, well, you see the steps, […] and then I consciously thought, when colleagues were taking the elevator, no, I'll take the stairs. (Q15: Medical personnel, development feedback, medium BMI)*

However, employees with high BMIs report negative emotions linked to receiving feedback on their health-related behavior:

*I recall that at some point we received an e-mail including norm groups [regarding activity levels] […] and then I really felt miserable, because I didn't fit in those groups. It was great for people who had high step counts, but for people with low step counts that wasn't nice at all. (Q16: Medical personnel, developmental feedback, high BMI)*

The advice they received as part of the developmental feedback was aimed at their work situation but, due to its general nature, it could also apply to their private situations, as reported by some employees noting that the “health responsibility” was being shifted from work to home. However, whereas employees with low and medium BMIs commented on this work-home shift in more neutral terms, employees with high BMIs were more negative:

*Well, when I had to get some groceries, I started to walk. And I'm taking the bicycle more often now, whenever I have to get something in our village. Before, I took the car, but I'm a lot more conscious about that now. (Q17: Medical personnel, development feedback, medium BMI)*

*Well, […] our whole company has to be healthy, and we all have to be good role models. […] And then I start thinking: What's next? Do I have to lose 20 kilograms of weight, because otherwise I can't work here? Because I'm not a good role model? (Q18: Medical personnel, developmental feedback, high BMI)*

This negative labeling of the attention to self-regulation of health-related behavior even in private time was projected onto the fitness opportunities that the employers provided after working hours: these are experienced as stigmatizing by employees with high BMIs. These employees indicate that they sometimes feel they are being watched and judged in their daily job, and feel as if the health programs offered by the employer after working hours are only fit for non-obese colleagues:

*I know I can join a company fitness class, […] but I'm afraid to do so. Because, who does that? All those trained bodies! I'm not going to stand amidst them, I really won't. (Q19: Medical personnel, developmental feedback, high BMI)*

*And then they are supporting ‘the week of taking the stairs’ […], but then, when I'm standing in front of the elevator, people tend to say “Oh, are you taking the elevator? We are taking the stairs!” That feels terrible. Really terrible. (Q20: Medical personnel, developmental feedback, high BMI)*

This supplementary analysis of additional data has provided some insight into the reasons why employees with high BMI respond differently to HSMA feedback than employees with lower BMI.

High BMI employees in the performance feedback group attach more salience to the provided norms and standards for healthy behavior, and experience more negative emotions when not reaching the norm, than employees with low BMIs. Further, they report that they increasingly notice limitations that stop them increasing their daily exercise.

Under the developmental feedback conditions, we see that both low and high BMI employees see more opportunities to change their workplace behavior, and both are aware that the responsibility for health at work to an extent shifts to the home environment. However, whereas employees with low BMIs comment about this shift in neutral terms, employees with high BMIs see this negatively. Further, the health promotion programs offered by the employer after working hours are frowned upon by those with high BMIs because they feel judged by these programs.

## Discussion

### Discussion of the Results

This study provides several new insights regarding the use of HSMAs in the workplace and their influence on employees' autonomy to regulate their own health-related behavior. We will first summarize the results of our study, after which we will discuss the theoretical and practical contributions. We also present some limitations and potential directions for future research.

This study shows that the use of HSMAs, such as the Fitbit, does not influence employees' perceived autonomy in self-regulating their health-related behavior at the workplace [i.e., their work health autonomy (WHA)], whereas it does reduce this perceived autonomy in the private situation, [i.e., home health autonomy (HHA)]. Looking at the effects of the type of feedback that participants received, we found that adding developmental feedback to performance feedback marginally enhanced the experienced WHA, but had no impact on HHA. Finally, we looked at the impact of using BMI as a single proxy for health status on these results, and we found that the effects of HSMAs on both WHA and HHA were negatively affected by BMI. That is, employees with a higher BMI suffered a greater loss of perceived autonomy in self-managing their health. Further, employees with a low BMI who received performance feedback experienced a relatively smaller loss of WHA than those with higher BMIs, and also reported an increase in HHA. The combined effects of feedback focus and BMI showed that the addition of developmental feedback mitigates the negative effects of HSMAs on WHA for employees with high BMIs, but at the same time decreases the HHA for these employees.

To better understand the influence of feedback focus and BMI interaction effects, we conducted additional interviews with participants with various BMIs. It showed that employees with high BMIs experienced, for several reasons, relatively less autonomy in self-regulating their health-related behavior in both the home and work situation. First, they tend to assign more salience to the general norms provided (i.e., walking 10,000 steps per day) than employees with lower BMIs. Employees with a low BMI experience the norm as a loose guideline, whereas people with a high BMI consider it as an important and strict norm that they are difficult to meet. When employees with high BMI then do not reach this norm, they experience negative emotions, and they express that they become increasingly aware of the limitations imposed by their surroundings that prevent them from reaching the norm. Further, employees with a low BMI consider healthy behavior part of their lifestyle whether at work or at home, whereas employees with a high BMI strictly separate these environments. As such, employees with high BMIs seem to allocate the feelings associated with receiving feedback from the HSMA to only one environment at a time, either at work or at home.

The present research has several implications for an appropriate and effective use of HSMAs, especially for users that are deemed less healthy. This is particularly of concern since HSMA-based workplace health programs are often implemented to specifically target these high risk groups. Our results do not confirm the general assumption underlying HSMAs that their use increases an individual's autonomy to self-regulate their health-related behavior (43, 44). Previous authors have suggested that while self-management tools may have the intention to “liberate” users, these, paradoxically, may impose autonomy (45). Using an HSMA as part of a workplace health promotion program tends to assume that users will feel autonomous and able to change behavior in a direction that is reflected in predefined norms set by health professionals (46). However, our empirical evidence indicates that users with a high BMI do not experience this elevated autonomy and are also likely to identify more issues that prevent them from optimally using the HSMAs. Our study is the first to observe this loss of perceived autonomy in an experimental setting, albeit that these findings are in line with findings reported by Puhl and Heuer (32) that obesity stigma impedes the effective use of public health interventions. The present results are also consistent with the felt fear for a loss of autonomy expressed by less healthy employees subject to preventive health measures by their employer (3).

Regarding feedback focus, our findings show that perceived autonomy is not automatically enhanced by providing developmental feedback (in addition to performance feedback usually provided by HSMAs), even though the literature suggests that its goal-setting and future-oriented nature should have positive effects on autonomy (20, 30). Interestingly, we also found that performance feedback alone was sufficient to increase the HHA of employees with low BMIs (see Figure 2), meaning that under certain conditions performance feedback can in itself be autonomy-enhancing. If we relate this to our initial ideas on perceived employee autonomy regarding health self-regulation, we see that these employees do not seem to feel as if autonomy is being imposed upon them (45), but rather that the direction in which the self-management information points them accords with their own beliefs, thereby increasing their capacity to autonomously change or continue their behavior.

The interaction effects of feedback focus and BMI suggest that participants with high BMIs attribute more salience to the norms implied by the HSMAs (e.g., 10,000 steps per day) and have more negative feelings about not reaching these norms than those with lower BMIs. This is in line with previous research on weight stigma and lifestyle changes indicating that overweight individuals have more difficulties in pursuing and persevering with lifestyle changes, potentially leading to greater self-stigmatization (31, 47). However, we saw that the addition of developmental feedback seems to mitigate the negative effects of HSMAs on WHA. This can be explained by the future-oriented and goal-setting nature of developmental feedback (20, 30), with feedback messages including concrete advice on how to alter ones' health-related behavior in the workplace, and tips on how to set and reach realistic goals through everyday actions.

These messages take away the experienced limitations in the workplace, because they actively offer a range of possibilities to exercise at work. Thereby, the negative emotions associated with the performance feedback are mitigated. Because this developmental feedback was focused on self-regulation of health behavior in the workplace and the performance feedback still highlighted that the employee did not meet the norms, the negative emotions about failing to meet the norms seem to be shifted from the workplace to home resulting in lower levels of HHA. Accordingly, high-BMI employees do not communicate with colleagues about their personal health goals, and do not seem to compensate for a lack of exercise in the workplace by additional exercise in the home environment. The differential findings for WHA and HHA for employees with high BMI confirm our initial idea that, in the case of workplace health promotion programs, autonomy regarding health self-regulation cannot be viewed as a single construct, but reflects the distinct aspects of WHA and HHA.

### Practical Implications

Our study shows that the use of HSMAs that are provided by the employer may cause harm for employees with high BMI, and that these harms may be mitigated by changing the type of feedback. Because the BMI of employees is a given factor when implementing a work health promotion program using HSMAs, we suggest that the negative effects of HSMAs should mainly be mitigated by thoughtful and inclusive implementation of these programs. Our study shows that HSMA usage can decrease employees' perceived autonomy to self-regulate their health-related behavior. In order to respect the autonomy of employees using HSMAs, the HSMA should not be a stand-alone tool but be embedded in a work health promotion program that enables employees to gradually change their behavior according to their own beliefs and change capacity. In our study, we saw that providing users with developmental feedback in addition to performance feedback is a step in the right direction, but also lifestyle coaching and flexible goal-setting could be considered as ways to increase the experienced feasibility of lifestyle changes for less healthy employees (2, 31), thereby increasing the autonomy of employees to pursue their health goals.

We also observed an increase in experienced stigma, which our high BMI respondents described as occurring because they experience an imbalance between attention to physical vs. mental health, and the use of general norms for healthy behavior instead of personalized norms and goals. The literature suggests these pitfalls can be avoided in both the development phase of health promotion programs, by including value levers in the design process (48), and the implementation phase, by using groups of employees and other stakeholders to address and evaluate (morally) relevant features and issues of the program (34).

Our study shows that employees with low BMIs benefit from performance feedback, but not from the additional developmental feedback. Therefore, we are less hesitant in recommending HSMAs for this group of employees, even if these HSMAs do not offer flexible goal-setting or other ways to personalize the feedback. We do however believe that employees with low BMIs may still benefit from additional personal coaching or supervision in altering their health-related behaviors because a low BMI does not necessarily equate to a healthy lifestyle.

### Limitations

Despite these relevant and interesting findings, this study has certain limitations that should be acknowledged. Given the nature of the HSMA, we have not been able to construct a control group that used the HSMA but did not receive feedback in addition to our two experimental groups. Since the HSMA gives continuous feedback, it is not possible to give some people a “placebo HSMA” since the lack of feedback would tell them immediately that they were in the placebo condition. Instead, we used a within-subjects design, comparing participants to their own pre-test characteristics. We have tried to limit the impact of the work environment as much as possible, by ensuring that work health promotion programs were not started, altered, or stopped during the experimental period.

The use of BMI as a proxy of health status in health research is much discussed (32, 49). For the present study, a relevant question is whether BMI sufficiently captures the differences in perceptions of health promotion interventions between individuals who consider themselves “healthy” or “unhealthy.” Health promotion interventions may be experienced very differently by individuals who feel like they only need to maintain their current health vs. individuals who face large behavioral changes in order to improve unhealthy conditions. A relevant question is whether BMI is a valid operationalization of these individual differences in health condition. We have adopted BMI as a suitable proxy of health because it has been proposed as a holistic measure of health, has high predictive validity across many health outcomes, is widely used in population and medical research, and can simply be self-reported by participants (33). Moreover, BMI is a relevant health factor for the self-regulation of the specific health-related behaviors (i.e., steps taken, stairs climbed, intensity of physical activities) we focused on in the present study. We do however share the concerns about the quality of BMI as an operationalization of people's health as discussed in literature (32, 49) and realize that its use is a limitation of the present research.

The HSMA that was used in the experiment showed the number of steps on the screen of the HSMA, thereby sending performance feedback by default. We therefore chose to send additional developmental feedback to the second experimental group, on top of the performance feedback that was similar to the feedback received by the first experimental group. This enabled us to evaluate the effects of additional developmental feedback. The effect of only receiving developmental feedback however has not been studied.

Regarding the given feedback and norms, the feedback was limited to the general norm of 10,000 steps per day (50). Although this norm is widely known and accepted in society, it is not without its critics in academia, and arguments are made to introduce other norms, such as the Active 10 (51). Our reason for using the 10,000 steps norm was that this norm is widely known throughout society, including to the vast majority of our study population, due to a large number of public health initiatives and the widespread availability of activity trackers.

Since the employees that participated in the experiment registered voluntarily, it is likely that these employees had an above-average interest in health and healthy behavior, or in changing their own lifestyle. This selection bias is however comparable with the selection bias that occurs when this type of workplace health promotion program is introduced in a regular working environment, because these programs are offered on a voluntary basis. Therefore, we believe this selection bias has no significant impact on the outcomes.

Since the experiment took place in a health care institution, there is a possibility that our participants had an idiosyncratic view on employee health and public health that is different from that of employees in other occupations performed in other types of organizations. However, given that the spread across the BMI spectrum in our sample is quite comparable with that of the average population (52), and the fact that 14% of the Dutch employees are employed in the health care industry (53), we do not think that the participants included in our sample would differ much from the general population in their responses to HSMAs and autonomy experiences. Notwithstanding, future research is needed to examine the generalizability of the present results to other occupations and other types of organizations.

### Areas for Future Research

The different effects of HSMA use on WHA and HHA for employees with high BMIs are hard to explain. The qualitative results suggest that employees with a high BMI make a clear distinction between their health-related behaviors at work and at home, whereas those with a lower BMI do not. Although we have not found other examples of this type of compartmentalization of health-related behavior, we believe this finding offers interesting insights into the workings of BMI, health, and lifestyle changes in the work environment, and we would recommend additional high-quality evaluative studies to further explore and explain these mechanisms.

In order to increase the likelihood of success in the use of employer-provided HSMAs, studies should further explore the effects of different types of feedback on employees. Our study shows that adding developmental feedback generates different reactions regarding perceived employee autonomy than when only performance-related feedback is offered. Future experiments might remove performance feedback and only offer developmental feedback, and might use different feedback media such as text messages, personal feedback, or an app with additional information. In this context, attention must be paid to the use of motivational techniques that are currently used in HSMAs (such as challenges with other persons, or publishing your data on social media) and the effect of these motivational techniques on the autonomy and privacy of the users.

## Conclusions

This article provides insights into the execution and outcomes of an experimental field study focused on the effects of HSMAs in the workplace. Using both quantitative data and information from a series of interviews, we have extended the understanding of employee autonomy regarding health self-regulation.

Generally, the use of HSMAs is viewed positively on the basis that they will enhance users' autonomy in self-regulating their behavior. However, our empirical study shows that this claim underlying the use of HSMAs at work is unjustified: the use of an HSMA does not significantly increase perceived autonomy, and even reduces it for less healthy employees. Nevertheless, the type of feedback usually given by HSMAs is not by definition harmful: the majority of the study population did not experience any negative effects from receiving only performance feedback. Developmental feedback can mitigate some of the negative effects shown among high-BMI participants, although it also transfers some of the negative effects to the home situation. These findings on the mitigation and transfer of the negative effects of HSMAs on the perceived autonomy of employees to self-regulate health-related behavior show a need for caution by employers, and reveal a need for further research on the responsible implementation of HSMAs in the workplace.

## Data Availability Statement

The datasets generated for this study are available on request to the corresponding author.

## Ethics Statement

The studies involving human participants were reviewed and approved by the Ethics Committee of the Faculty of Economics and Business at the University of Groningen. The participants provided their written informed consent to participate in this study.

## Author Contributions

AB designed the experiment and collected the data. AB and OJ analyzed the quantitative data. Interview protocols were drawn by AB, EM, and MB. Analysis of the interview data was done by AB and MB. AB drafted the manuscript under supervision from JW, OJ, EM, and MB. All authors contributed to the article and approved the submitted version.

## Conflict of Interest

The authors declare that the research was conducted in the absence of any commercial or financial relationships that could be construed as a potential conflict of interest.

## References

[1] De Jongh J Mcdougal F. Gezonde Leefstijl en Vitaliteit op de Werkvloer: Een Kwantitatief Onderzoek Onder Werknemers in Opdracht van Convenant Gezond Gewicht. Amsterdam. (2014). Available online at: https://www.stvda.nl/~/media/Files/Stvda/Thema/Arbeidsomstandigheden/vitaliteit/20140925-ruigrok-Netpanel.ashx (accessed Dec 6, 2017).

[2] Hendriksen IJM Snoijer M De Kok BPH van Vilsteren J Hofstetter H. Effectiveness of a multilevel workplace health promotion program on vitality, health, and work-related outcomes. J Occup Environ Med. (2016) 58:575–83. 10.1097/JOM.0000000000000747 27136605 PMC4883645

[3] Damman OC van der Beek AJ Timmermans DRM. Employees are ambivalent about health checks in the occupational setting. Occup Med. (2015) 65:451–8. 10.1093/occmed/kqv048 26023107

[4] Blake H Batt ME. Employee perceptions of a pedometer walking intervention in a hospital workplace. Int J Heal Promot Educ. (2015) 53:257–70. 10.1080/14635240.2015.1016621

[5] Husain I Spence D. Can healthy people benefit from health apps? BMJ. (2015) 350:h2520. 10.1136/bmj.h2520 25873345

[6] Malik SH Blake H Suggs LS. A systematic review of workplace health promotion interventions for increasing physical activity. Br J Health Psychol. (2014) 19:149–80. 10.1111/bjhp.12052 23827053

[7] Grady PA Gough LL. Self-management: a comprehensive approach to management of chronic conditions. Am J Public Health. (2014) 104:25–31. 10.2105/AJPH.2014.302041 24922170 PMC4103232

[8] Schopp LH Clark MJ Lamberson WR Uhr DJ Minor MA. A randomized controlled trial to evaluate outcomes of a workplace self-management intervention and an intensive monitoring intervention. Health Educ Res. (2017) 32:219–32. 10.1093/her/cyx042 28486643

[9] Ryan RM Deci EL. Self-determination theory and the facilitation of intrinsic motivation, social development, and well-being. Am Psychol. (2000) 55:68–78. 10.1037/0003-066X.55.1.68 11392867

[10] Ryan RM Deci EL. Self-regulation and the problem of human autonomy: does psychology need choice, self-determination, and will? J Pers. (2006) 74:1557–86. 10.1111/j.1467-6494.2006.00420.x 17083658

[11] Silva MN Vieira PN Coutinho SR Minderico CS Matos MG Sardinha LB. Using self-determination theory to promote physical activity and weight control: a randomized controlled trial in women. J Behav Med. (2010) 33:110–22. 10.1007/s10865-009-9239-y 20012179

[12] Borg J Merom D Rissel C. Staff walking program: a quasi-experimental trial of maintenance newsletters to maintain walking following a pedometer program. Heal Promot J Aust. (2010) 21:26–32. 10.1071/HE10026 20406149

[13] Alder GS. Ethical issues in electronic performance monitoring: a consideration of deontological and teleological perspectives. J Bus Ethics. (1998) 17:729–43. 10.1023/A:1005776615072

[14] Alder GS Ambrose ML. An examination of the effect of computerized performance monitoring feedback on monitoring fairness, performance, and satisfaction. Organ Behav Hum Decis Process. (2005) 97:161–77. 10.1016/j.obhdp.2005.03.003

[15] Brey P. Worker autonomy and the drama of digital networks in organizations. J Bus Ethics. (1999) 22:15–25. 10.1023/A:1006199816737

[16] Leclercq-Vandelannoitte A. An ethical perspective on emerging forms of ubiquitous IT-based control. J Bus Ethics. (2017) 142:139–54. 10.1007/s10551-015-2708-z

[17] Martin K Freeman RE. Some problems with employee monitoring. J Bus Ethics. (2003) 43:353–61. 10.1023/A:1023014112461

[18] Campbell JI Eyal N Musiimenta A Haberer JE. Ethical questions in medical electronic adherence monitoring. J Gen Intern Med. (2016) 31:338–42. 10.1007/s11606-015-3502-4 26358284 PMC4762813

[19] Owens J Cribb A. “My Fitbit Thinks I Can Do Better!” do health promoting wearable technologies support personal autonomy? Philos Technol. (2017) 32:23–38. 10.1007/s13347-017-0266-2

[20] Li N Harris TB Boswell WR Xie Z. The role of organizational insiders' developmental feedback and proactive personality on newcomers' performance: an interactionist perspective. J Appl Psychol. (2011) 96:1317–27. 10.1037/a0024029 21688879

[21] Bravata DM Smith-Spangler C Sundaram V Gienger AL Lin N Lewis R. Using pedometers to increase physical activity and improve health. JAMA. (2007) 298:2296. 10.1001/jama.298.19.2296 18029834

[22] Chatzisarantis NLD Hagger MS. Effects of an intervention based on self-determination theory on self-reported leisure-time physical activity participation. Psychol Heal. (2009) 24:29–48. 10.1080/08870440701809533 20186638

[23] Fortier MS Sweet SN O'sullivan TL Williams GC. A self-determination process model of physical activity adoption in the context of a randomized controlled trial. Psychol Sport Exerc. (2007) 8:741–57. 10.1016/j.psychsport.2006.10.006

[24] Rose EA Parfitt G Williams S. Exercise causality orientations, behavioural regulation for exercise and stage of change for exercise: exploring their relationships. Psychol Sport Exerc. (2005) 6:399–414. 10.1016/j.psychsport.2004.07.002

[25] Williams GC Mcgregor HA Zeldman A Freedman ZR Deci EL. Testing a self-determination theory process model for promoting glycemic control through diabetes self-management. Heal Psychol. (2004) 23:58–66. 10.1037/0278-6133.23.1.58 14756604

[26] Williams GC Ne Gagné M Ryan RM Deci EL. Facilitating autonomous motivation for smoking cessation. Heal Psychol. (2002) 21:40–50. 10.1037/0278-6133.21.1.40 11846344

[27] Deci EL Vallerand RJ Pelletier LG Ryan RM. Motivation and education: the self-determination perspective. Educ Psychol. (1991) 26:325–46. 10.1207/s15326985ep2603&4_6

[28] Braukmann J Schmitt A Duranová L Ohly S. Identifying ICT-related affective events across life domains and examining their unique relationships with employee recovery. J Bus Psychol. (2018) 33:529–44. 10.1007/s10869-017-9508-7

[29] Kluger AN DeNisi A. The effects of feedback interventions on performance: a historical review, a meta-analysis, and a preliminary feedback intervention theory. Psychol Bull. (1996) 119:254–84. 10.1037/0033-2909.119.2.254

[30] Zhou J. When the presence of creative coworkers is related to creativity: role of supervisor close monitoring, developmental feedback, and creative personality. J Appl Psychol. (2003) 88:413–22. 10.1037/0021-9010.88.3.413 12814291

[31] Delahanty LM Meigs JB Hayden D Williamson DA Nathan DM. Psychological and behavioral correlates of baseline BMI in the Diabetes Prevention Program (DPP). Diabetes Care. (2002) 25:1992–8. 10.2337/diacare.25.11.1992 12401745 PMC1475806

[32] Puhl RM Heuer CA. Obesity stigma: important considerations for public health. Am J Public Health. (2010) 100:1019–28. 10.2105/AJPH.2009.159491 20075322 PMC2866597

[33] Gutin I. In BMI we trust: reframing the body mass index as a measure of health. Soc Theory Heal. (2018) 16:256–71. 10.1057/s41285-017-0055-0 31007613 PMC6469873

[34] Ten Have M van Der Heide A Mackenbach JP De Beaufort ID. An ethical framework for the prevention of overweight and obesity: a tool for thinking through a programme's ethical aspects. Eur J Public Health. (2012) 23:299–305. 10.1093/eurpub/cks052 23132871

[35] Madsen SR. The effects of home-based teleworking on work-family conflict. Hum Resour Dev Q. (2003) 14:35–58. 10.1002/hrdq.1049

[36] Robroek SJW van Lenthe FJ van Empelen P Burdorf A. Determinants of participation in worksite health promotion programmes: a systematic review. Int J Behav Nutr Phys Act. (2009) 6:1–12. 10.1186/1479-5868-6-26 19457246 PMC2698926

[37] Eysenbach G. The law of attrition. J Med Internet Res. (2005) 7:1–9. 10.2196/jmir.7.1.e11 15829473 PMC1550631

[38] Norman GJ Zabinski MF Adams MA Rosenberg DE Yaroch AL Atienza AA. A review of ehealth interventions for physical activity and dietary behavior change. Am J Prev Med. (2007) 33:336–45. 10.1016/j.amepre.2007.05.007 17888860 PMC2180189

[39] Hackman JR Oldham GR. The Job Diagnostic Survey: an Instrument for the Diagnosis of Jobs and the Evaluation of Job Redesign Projects. Yale Univ New Haven CT Dept of Administrative Sciences (1974).

[40] Hackman JR Oldham GR. Work Redesign. Reading, MA: Addison-Wesley (1980).

[41] Krippendorff K. Reliability in content analysis. Hum Commun Res. (2004) 30:411–33. 10.1111/j.1468-2958.2004.tb00738.x

[42] Becker TE. Potential problems in the statistical control of variables in organizational research: a qualitative analysis with recommendations. Organ Res Methods. (2005) 8:274–89. 10.1177/1094428105278021

[43] Karapanos E Gouveia R Hassenzahl M Forlizzi J. Wellbeing in the making: peoples' experiences with wearable activity trackers. Psychol Well Being. (2016) 6:4. 10.1186/s13612-016-0042-6 27376017 PMC4908170

[44] Nelson EC Verhagen T Noordzij ML. Health empowerment through activity trackers: an empirical smart wristband study. Comput Human Behav. (2016) 62:364–74. 10.1016/j.chb.2016.03.065

[45] Kendall E Ehrlich C Sunderland N Muenchberger H Rushton C. Self-managing versus self-management: reinvigorating the socio-political dimensions of self-management. Chronic Illn. (2011) 7:87–98. 10.1177/1742395310380281 20921037

[46] Lowenberg JS. Health promotion and the “Ideology of Choice.” Public Health Nurs. (1995) 12:319–23. 10.1111/j.1525-1446.1995.tb00155.x 7479540

[47] Puhl RM Brownell KD. Ways of coping with obesity stigma: review and conceptual analysis. Eat Behav. (2003) 4:53–78. 10.1016/S1471-0153(02)00096-X 15000988

[48] Shilton K. Values levers: building ethics into design. Sci Technol Hum Values. (2013) 38:374–97. 10.1177/0162243912436985

[49] MacLean L Edwards N Garrard M Sims-Jones N Clinton K Ashley L. Obesity, stigma and public health planning. Health Promot Int. (2009) 24:88–93. 10.1093/heapro/dan041 19131400

[50] Johnman C Mackie P Sim F. 10,000 steps into the digital age. Public Health. (2017) 149:A1–3. 10.1016/j.puhe.2017.06.018 28709683

[51] Brannan MG Foster CE Timpson CM Clarke N Sunyer E Amlani A. Active 10 – a new approach to increase physical activity in inactive people in England. Prog Cardiovascu Dis. (2019) 62:135–9. 10.1016/j.pcad.2019.02.001 30796943

[52] Centraal Bureau voor de Statistiek and Rijksinstituut voor Volksgezondheid en Milieu. Leefstijlmonitor. (2020). Available online at: https://www.rivm.nl/leefstijlmonitor/gezond-gewicht (accessed May 22, 2020).

[53] Centraal Bureau voor de Statistiek. Statline: Werkgelegenheid in de zorg en welzijn; baankenmerken. (2020). Available online at: https://azwstatline.cbs.nl/?dl=1B174#/AZW/nl/dataset/24047NED/table (accessed May 22, 2020).

