# Supplementary material for: Health Self-Management Applications in the Work Environment: The Effects on Employee Autonomy
Source: Front Digit Health. 2020 Jul 17;2:9. doi: 10.3389/fdgth.2020.00009 (PMC8521808; doi:10.3389/fdgth.2020.00009)
Supplement: Supplementary file 1 [file Data_Sheet_1.docx]

**Appendix 1:** Examples of feedback messages

*Performance feedback condition*

Feedback on request (available through the Fitbit One):

- Current daily step count
- Current number of stairs taken
- Estimated number of calories burned today
- Estimated distance walked today

Feedback by e-mail (sent on average once a week):

- Daily step count for the last 7 days
- Daily number of stairs taken for the last 7 days
- Number of minutes per day of daily activity (low, medium or high intensity)

*Developmental feedback condition*

Feedback on request (available through the Fitbit One):

- Current daily step count
- Current number of stairs taken
- Estimated number of calories burned today
- Estimated distance walked today

Feedback by e-mail (sent on average once a week):

- Daily step count for the last 7 days
- Daily number of stairs taken for the last 7 days
- Number of minutes per day of daily activity (low, medium or high intensity)

Added in week 1:

- Information on low, medium and high intensity activity
  - Feedback on activity levels
  - Advice on how to alter activity levels
  - Link to website with more information about these activity levels

Added in week 2:

- Information on medium intensity activity and increasing physical activity
  - Feedback on medium intensity activity level
  - Information about activities that are of medium intensity
  - Advice on how to set goals and reach goals regarding physical activity levels

Added in week 3:

- Information on high intensity activity and exercising together
  - Feedback on high intensity activity level
  - Information on how exercising with others can affect and improve behavior
  - Link to website where people can find a ‘Beweegmaatje’ (someone to exercise with)

Added in week 4:

- Information on continuing behavioral change
  - Feedback on activity levels
  - Information on how to persist behavioral change
  - Mitigation strategies to avoid risks that keep one from exercising
